# Contemporary data on treatment practices for low-density lipoprotein cholesterol in 3867 patients who had suffered an acute coronary syndrome across the world

**DOI:** 10.1016/j.dib.2017.11.034

**Published:** 2017-11-14

**Authors:** Anselm K. Gitt, Dominik Lautsch, Jean Ferrières, Gaetano M. De Ferrari, Ami Vyas, Carl A. Baxter, Lori D. Bash, Veronica Ashton, Martin Horack, Wael Almahmeed, Fu-Tien Chiang, Kian Keong Poh, Philippe Brudi, Baishali Ambegaonkar

**Affiliations:** a Herzzentrum Ludwigshafen, Germany; b Institut für Herzinfarktforschung Ludwigshafen, Germany; c Merck & Co., Inc., Kenilworth, NJ, USA; d Rangueil Hospital, Toulouse University School of Medicine, Toulouse, France; e Department of Molecular Medicine University of Pavia, and Cardiac Intensive Care Unit and Laboratories for Experimental Cardiology, IRCCS Fondazione Policlinico San Matteo, Pavia, Italy; f Rutgers University, School of Public Health, Piscataway, NJ, USA; g MSD Ltd. Hoddesdon, UK; h Agile-1 for Merck & Co., Inc., Kenilworth, NJ, USA; i Sheikh Khalifa Medical City, Abu Dhabi, UAE; j Heart and Vascular Institute, Cleveland Clinic Abu Dhabi, UAE; k National Taiwan University Hospital, Taipei, Taiwan and Fu-Jen Catholic University Hospital, Taipei, Taiwan; l Yong Loo Lin School of Medicine, National University of Singapore, Singapore; m Department of Cardiology, National University Heart Center Singapore, National University Health System, Singapore

**Keywords:** Low-density lipoprotein cholesterol, Treatment target, Global, Region, Statins

## Abstract

DYSIS II ACS was a longitudinal, observational study in 3867 patients from 18 countries. They were being hospitalized after suffering an acute coronary syndrome. Evaluations were performed at the time of admission and again 120±15 days following the date of admission (the follow-up time point). 2521 patients were on active lipid lowering treatment (LLT) at admission. Mean atorvastatin dose was 22 mg per day and 2.7% received ezetimibe in combination with a statin. At discharge from hospital, 3767 patients received LLT expressed as a mean atorvastatin dose of 36 mg per day with 4.8% receiving ezetimibe on top of a statin. After 120 days, intensity in lipid lowering treatment was reduced to 32 mg per day with 4.9% of the patients receiving ezetimibe and a statin. Of note, during this 4-month follow up period, only 32% of all patients received laboratory lipid testing. 37% attained the low density lipoprotein cholesterol (LDL-C) target value of <70 mg/dl after 120 days. There are differences in the therapy administered as well as in the switch strategies when comparing the data from the respective countries studied.

**Conclusions:**

Only one in three patients achieved the LDL-C target value following only marginal improvements in atorvastatin dose or combination therapy after an ACS event.

**Specifications Table**

**Table:** 

Subject area	Biology
More specific subject area	Dyslipidemia and cardiovascular risk
Type of data	Tables and Figures
How data was acquired	Worldwide survey
Data format	Analyzed
Experimental factors	Observational, longitudinal registry
Experimental features	Comparison of lipid lowering therapies administered in patients post acute coronary syndrome, as well as LDL-C target achievement.
Data source location	Institut für Herzinfarktforschung, Ludwigshafen, Germany
Data accessibility	Data are included in this article

**Value of the data**

• These data have been collected under real life conditions across the world.
• Stratification per country can help to facilitate a scientific dialogue for the benefit of coronary patients in these countries, but also help to compare treatment standards between geographies of the world.
• The data presented can help to guide treatment decisions for novel lipid lowering agents.

## 1 Data

**Fig. 1:**
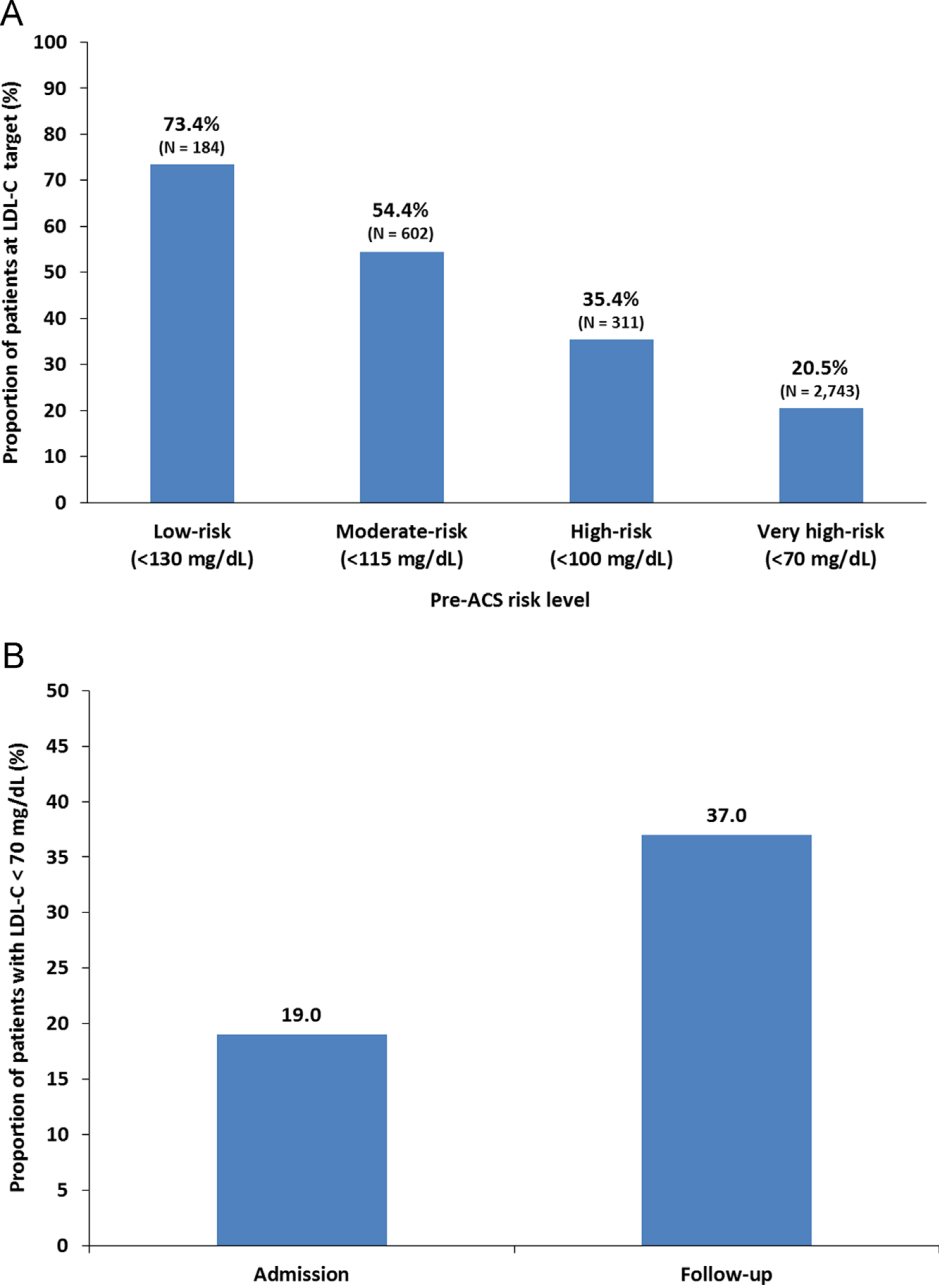
LDL-C target attainment for ACS cohort. (A) LDL-C target attainment by pre-ACS risk level (ESC/EAS guidelines); (B) Proportion of ACS patients with an LDL-C level of <70 mg/dL at hospital admission and at 120-day follow-up (for patients with values available at both time points, N = 1071).

**Fig. 2:**
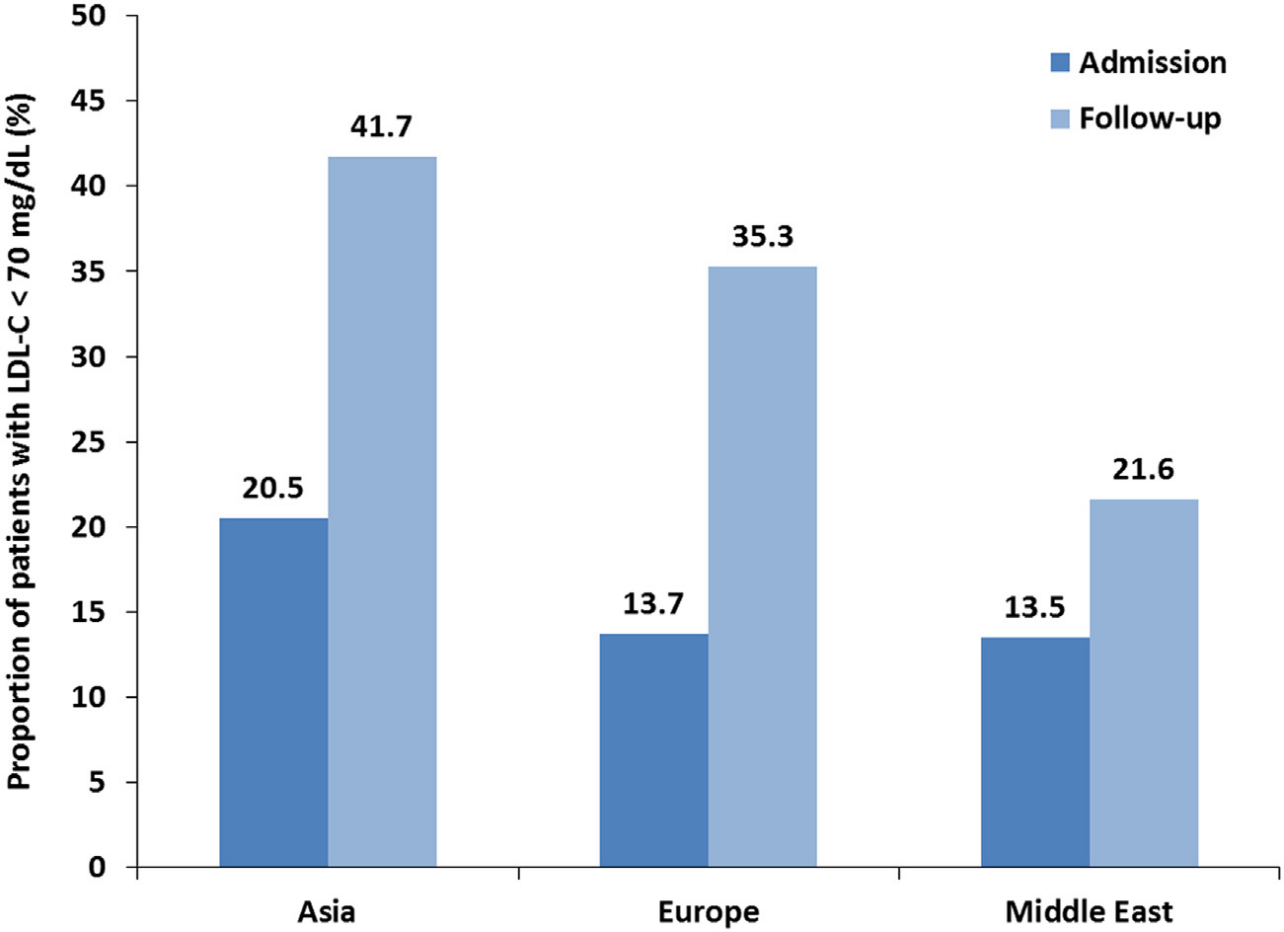
LDL-C target value attainment by region. Target value attainment rates were calculated for the 1,071 patients with LDL-C data at both time points.

See Fig. 1, Fig. 2 and Table 1, Table 2, Table 3.

**Table 1:** Indicates the change in lipid-lowering therapy at admission to a hospital for the treatment of an ACS, as well as the changes applied during hospital stay, at discharge and after a 120 day follow up period.

		At admission			During hospital stay			At discharge			120 days post ACS					
	*N*	LLT (%)	AED	E/S	LLT (%)	AED	E/S	% LLT	AED	E/S	*N*	% LLT	AED	E/S	Lipid lab during follow up (%)	LDL-C<70 mg/dl at follow up (%)
Egypt	*199*	147 (26.1)	29.72	0 (0.0)	199 (100.0)	54.02	0 (0.0)	199 (100)	53.12	0 (0.0)	*151*	148 (98.0)	45.74	0 (0.0)	23.8%	5.6%
France	*468*	277 (40.8)	21.72	20 (4.3)	450 (96.2)	48.02	14 (3.0)	453 (96.8)	48.86	14 (3.0)	*326*	312 (95.7)	40.81	15 (4.6)	48.5%	50.6%
Germany	*461*	270 (41.4)	18.42	14 (3.0)	447 (97.0)	20.59	19 (4.1)	447 (97.0)	20.91	23 (5.0)	*390*	357 (91.5)	21.83	21 (5.4)	27.9%	19.3%
Greece	*200*	159 (20.5)	20.31	12 (6.0)	196 (98.0)	31.28	9 (4.5)	196 (98.0)	31.18	12 (6.0)	*194*	188 (96.9)	29.87	15 (7.7)	44.3%	22.1%
Hong Kong	*140*	67 (52.1)	13.86	1 (0.7)	135 (96.4)	16.46	1 (0.7)	131 (93.6)	17.36	1 (0.7)	*136*	132 (97.1)	17.18	0 (0.0)	56.6%	48.1%
India	*521*	404 (22.5)	28.51	2 (0.4)	519 (99.6)	45.54	1 (0.2)	510 (97.9)	43.4	0 (0.0)	*513*	482 (94.0)	37.01	0 (0.0)	15.6%	57.5%
Ireland	*57*	32 (43.9)	32.67	1 (1.8)	55 (96.5)	66.98	0 (0.0)	57 (100)	62.46	1 (1.8)	*56*	56 (100.0)	60	1 (1.8)	57.1%	46.9%
Italy	*212*	142 (33)	26.71	9 (4.2)	207 (97.6)	57.84	3 (1.4)	206 (97.2)	56.04	9 (4.2)	*171*	168 (98.2)	50.55	11 (6.4)	31.6%	37.0%
Jordan	*40*	24 (40)	24.35	1 (2.5)	39 (97.5)	35.26	5 (12.5)	40 (100)	35.13	7 (17.5)	*30*	30 (100)	37.33	0 (0.0)	3.3%	100.0%
Lebanon	*82*	61 (25.6)	24.49	1 (1.2)	82 (100)	55.98	1 (1.2)	82 (100)	45.18	2 (2.4)	*78*	76 (97.4)	38.42	1 (1.3)	28.2%	27.3%
Philippines	*48*	26 (45.8)	48.27	0 (0.0)	46 (95.8)	60.22	0 (0.0)	47 (97.9)	56.74	0 (0.0)	*21*	21 (100)	44.5	0 (0.0)	19.0%	50.0%
Saudi Arabia	*150*	140 (6.7)	23.79	18 (12.0)	150 (100)	59.8	26 (17.3)	150 (100)	38.13	64 (42.7)	*150*	150 (100)	40.27	55 (36.7)	15.3%	8.7%
Singapore	*126*	84 (33.3)	19.37	2 (1.6)	121 (96)	35.37	4 (3.2)	122 (96.8)	35.29	4 (3.2)	*108*	106 (98.1)	33.89	3 (2.8)	61.1%	34.8%
South Korea	*308*	162 (47.4)	16.91	11 (3.6)	300 (97.4)	22.57	34 (11.0)	306 (99.4)	22.47	36 (11.7)	*301*	296 (98.3)	20.72	27 (9.0)	26.2%	62.0%
Taiwan	*130*	58 (55.4)	14.42	4 (3.1)	114 (87.7)	18.58	2 (1.5)	113 (86.9)	17.69	3 (2.3)	*123*	97 (78.9)	18.47	3 (2.4)	33.3%	36.6%
Thailand	*320*	188 (41.3)	16.9	6 (1.9)	317 (99.1)	27.92	6 (1.9)	311 (97.2)	28.07	7 (2.2)	*285*	275 (96.5)	28.2	11 (3.9)	51.9%	25.7%
UAE	*200*	129 (35.5)	23.51	1 (0.5)	199 (99.5)	40.43	5 (2.5)	197 (98.5)	40.1	4 (2.0)	*166*	161 (97.0)	39.28	1 (0.6)	17.5%	44.8%
Vietnam	*205*	151 (26.3)	17.4	1 (0.5)	204 (99.5)	21.84	0 (0.0)	200 (97.6)	19.93	0 (0.0)	*191*	186 (97.4)	18.49	1 (0.5)	13.6%	26.9%
All	*3867*	2521 (34.8)	22.36	104 (2.7)	3780 (97.8)	37.43	130 (3.4)	3767 (97.4)	35.88	187 (4.8)	*3390*	3241 (95.6)	32.41	165 (4.9)	31.6%	37.0%

LLT, Lipid lowering treatment; AED, Atorvastatin equivalent dose; E/S, ezetimibe in combination with any statin: ACS, acute coronary syndrome; LDL-C, low density lipoprotein cholesterol; UAE, United Arab Emirates. % target value attainment after 120 days follow up given for those patients with recent lab values available.

**Table 2:** Predictors of LDL-C target value attainment among treated ACS patients.

	Full model			Stepwise model		
	OR	95% CI	P value	OR	95% CI	P value
Age ≥70	1.20	0.96–1.50	0.109	–	–	–
Female	0.60	0.47–0.77	<0.001	0.61	0.48–0.78	<0.001
BMI >30 kg/m^2^	0.63	0.49–0.81	<0.001	0.61	0.48–0.78	<0.001
Current smoking	0.59	0.44–0.77	<0.001	0.57	0.43–0.74	<0.001
Sedentary lifestyle	0.90	0.73–1.10	0.293	–	–	–
Stable angina	0.88	0.65–1.20	0.418	–	–	–
Chronic kidney disease	1.52	1.09–2.13	0.014	1.58	1.14–2.20	0.006
Type 2 diabetes mellitus	1.33	1.08–1.64	0.007	1.31	1.07–1.61	0.009
History of chronic heart failure	1.38	0.99–1.94	0.060	1.40	1.00–1.96	0.050
Hypertension	0.98	0.77–1.25	0.881	–	–	–
Statin dose (atorvastatin dose equivalent, mg/day)	1.01	1.004–1.016	<0.001	1.009	1.004–1.015	0.001

BMI, body mass index; CI, confidence interval; OR, odds ratio.

**Table 3:** Regional differences in lipid-lowering therapy.

	Admission (N = 3867)				Follow-up (N = 3558)			
	Asia	Europe	Middle East	P value	Asia	Europe	Middle East	P value
Statin monotherapy	58.8%	53.8%	69.5%	<0.001	89.8%	83.1%	85.4%	<0.001
Non-statin monotherapy	1.0%	2.4%	0.6%	<0.001	0.4%	0.7%	0.2%	N.S.
Statin+ezetimibe	1.5%	4.0%	3.1%	<0.001	2.7%	5.5%	9.9%	<0.001
Statin+other non-statin	2.1%	2.8%	1.5%	N.S.	2.2%	5.8%	2.8%	<0.001
Not treated	36.6%	37.1%	25.3%	<0.001	4.4%	4.9%	1.7%	<0.001
Atorvastatin dose equivalent, mean±SD mg/day^a^	22±18	22±17	25±14	<0.001	27±18	35±25	41±18	<0.001

SD, standard deviation; N.S., not significant.

a In statin treated patients (N = 2466 at admission; N = 3226 at follow up 120 days after the ACS).

## 2 Experimental design, materials and methods

DYSIS II ACS was a multicenter, longitudinal, observational study that included 3867 patients from 18 countries in Europe, the Middle East, South-, Southeast- and East-Asia.

The study was approved by the relevant ethics committees and carried out in agreement with local laws.

Inclusion criteria were as follows: 1) provision of written informed consent, 2) aged ≥18, 3) hospitalized for an ACS in 2013–2014, 4) availability of a full fasting or non fasting lipid profile based on blood drawn within 24 hours of admission, and 5) not participating in a clinical trial. Data were assessed at presentation for an acute coronary syndrome (ACS), at hospital stay and discharge, as well as after a predefined period of 120 days.

The ESC/EAS dyslipidemia guidelines (2011) were used as a reference in order to determine target value attainment after 120 days [1]. Low density lipoprotein (LDL-C) treatment target thus was <70 mg/dl. Since the guidelines also highlight the use of statins in highest tolerated dose, followed by the use of lipid lowering combination therapy, we determined statin dose administered, calculated as atorvastatin equivalent doses [2].

Data were collected in an electronic case report form and processed in a central web-based database at the Institut für Herzinfarktforschung, Ludwigshafen, Germany. It was used for both collection and storage of the data.

SAS version 9.3 (Cary, NC, USA) was used for performing the calculations. Data are presented as absolute numbers and percentages (*n*/*N*), pertaining to either the baseline sample or the 3,390 patients who presented for the follow up visit.
