# Supplementary material for: Contemporary data on treatment practices for low-density lipoprotein cholesterol in 3867 patients who had suffered an acute coronary syndrome across the world
Source: Data Brief. 2017 Nov 14;16:369–75. doi: 10.1016/j.dib.2017.11.034 (PMC5723270; doi:10.1016/j.dib.2017.11.034)
Supplement: Supplementary file 1 — Supplementary material [file mmc1.pdf]

AUTHOR DECLARATION DIB-D-17-00785

We wish to draw the attention of the Editor to the following facts which may be considered as potential conflicts of interest and to significant financial contributions to this work.

> *A.K.G. received honoraria from Merck &Co., Inc. for contributions to the DYSIS study. M. H. reports that his institution received funding for recruitment and biostatistics for the DYSIS registry. J.F. received grants and personal fees from Amgen, Merck & Co., Inc., and Sanofi. G.M.D.F. received speaker's fee from Merck & Co., Inc., and Amgen. W.A. received honorarium from Merck & Co., Inc. At the time of the study, A.V. was a full-time employee of Rutgers Uni- versity, which received grant funding for this project from Merck & Co., Inc. A.V. is currently employed by the University of Rhode Is- land. V.A. was a full-time contractor of Agile-1 for Merck & Co., Inc. B.A., C.B., P.B., D.L. and L.D.B. are employees of Merck & Co. Inc., or MSD, a subsidiary of Merck & Co., Inc., Kenilworth, NJ, USA. F.T.C. and K.K.P. have no interests to disclose.*

We confirm that the manuscript has been read and approved by all named authors and that there are no other persons who satisfied the criteria for authorship but are not listed. We further confirm that the order of authors listed in the manuscript has been approved by all of us.

We confirm that we have given due consideration to the protection of intellectual property associated with this work and that there are no impediments to publication, including the timing of publication, with respect to intellectual property. In so doing we confirm that we have followed the regulations of our institutions concerning intellectual property.

We further confirm that any aspect of the work covered in this manuscript that has involved either experimental animals or human patients has been conducted with the ethical approval of all relevant bodies and that such approvals are acknowledged within the manuscript.

We understand that the Corresponding Author is the sole contact for the Editorial process (including Editorial Manager and direct communications with the office). He/she is responsible for communicating with the other authors about progress, submissions of revisions and final approval of proofs. We confirm that we have provided a current, correct email address which is accessible by the Corresponding Author and which has been configured to accept email from the journal.

Signed by Dr. Dominik Lautsch on behalf of all authors:

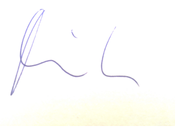

Vienna, Oct 31 2017
